# Real-time cholesterol sorting in *Plasmodium falciparum*-erythrocytes as revealed by 3D label-free imaging

**DOI:** 10.1038/s41598-020-59552-9

**Published:** 2020-02-17

**Authors:** Eri H. Hayakawa, Kentaro Yamaguchi, Masahiro Mori, Glenn Nardone

**Affiliations:** 1 Division of Medical Zoology, Department of Infection and Immunity, Jichi Medical University, Yakushiji 3311-1, Shimotsuke, Tochigi 329-0498 Japan; 2 LMS Co., Ltd., 3-6-7, Hongo, Bunkyo-ku, Tokyo 113-0033 Japan; 3 Present Address: BioTek Japan, llc., 2-7-1-8F Taito, Taito-ku, Tokyo 110-0016 Japan; 4 Faculty of Pharmaceutical Sciences, Chiba Institute of Science, 15-8 Choshi, Chiba, 288-0025 Japan; 5 Research Technology Branch, National Institute of Allergy and Infectious Diseases, National Institutes of Health, Bethesda, MD 20892-5766 USA

**Keywords:** Parasite biology, Structural biology

## Abstract

Cholesterol, a necessary component of animal cell membranes, is also needed by the lethal human malaria parasite *Plasmodium falciparum*. Because *P. falciparum* lacks a cholesterol synthesis pathway and malaria patients have low blood cholesterol, we speculated that it scavenges cholesterol from them in some way. We used time-lapse holotomographic microscopy to observe cholesterol transport in live *P. falciparum* parasites and structurally investigate erythrocyte membranes, both during and after *P. falciparum* invasion of human erythrocytes. After *P. falciparum* initially acquired free cholesterol or inner erythrocytic membrane-derived cholesterol, we observed budding lipid membranes elongating into the cytosol and/or membrane segments migrating there and eventually fusing with the parasite membranes, presumably at the parasitophorous vacuole membrane (PVM). Finally, the cholesterol-containing segments were seen to surround the parasite nucleus. Our imaging data suggest that a novel membrane transport system operates in the cytosol of *P. falciparum*-infected erythrocytes as a cholesterol import system, likely between the PVM and the erythrocyte membrane, and that this transportation process occurs during the live erythrocyte stages of *P. falciparum*.

## Introduction

Malaria, an infectious disease caused by *Plasmodium* species (*spp*.), still retains its prevalence in tropical and subtropical regions of the world, particularly in Africa and East South Asia. RTS,S/AS01, the first *Plasmodium falciparum* vaccine recommended by World Health Organization (https://www.who.int/malaria/media/malaria-vaccine-implementation-qa/en/), is somewhat effective for young children aged 5–17 months. Furthermore, RTS,S/AS01 was shown to be an effective control measure in a malaria endemic area of Africa^1^. However, with no fully protective vaccine for adults, and the possible emergence of vaccine-resistant malaria in the future, it remains important to gain better understanding of malarial infections and to clarify the biology of the pathogen in its host. After a *Plasmodium spp*.-infected mosquito bites the host’s skin, parasites first infect the liver where they mature and multiply, eventually rupturing the cells and emerging as merozoites primed to invade erythrocytes where they proceed to grow and multiply by asexual reproduction before rupturing the cells and invading new ones. Of the five species of malaria parasites that can infect humans^2^, the most lethal malaria parasite is *P. falciparum*, which causes the most severe disease, the manifestations of which can include cerebral infarct, severe anemia, and coma, and the highest number of fatalities. During the infection process in humans, the parasite’s lifecycle imposes structural and physico-chemical modifications on host erythrocytes. Such modifications involve the transportation of parasite proteins to the surface of the erythrocyte and the modification of lipid raft structures on the erythrocyte membrane^3,4^. These changes alter the erythrocyte’s surface structure and contribute to the clinical manifestations of the disease^4–7^. Furthermore, people who become infected with *P. falciparum* parasites commonly develop decreased blood levels of low-density lipoprotein (LDL), high-density lipoprotein (HDL) and cholesterol^8,9^. Such alterations in blood constituents suggest that *P. falciparum* may use various lipid components in the blood, including lipoproteins, for its survival.

In eukaryotic cells, intracellular lipid transport utilizes vesicle transport (COP I transport vesicles)^10^ and non-vesicle transport systems (e.g., flip-flop^11,12^, lateral exchange^13,14^, and lipid transfer proteins^15^). Cholesterol, an essential lipid for eukaryotic cells, is typically sorted in the endoplasmic reticulum and is sent to organelles and plasma membranes^16–18^. However, unlike other eukaryotic cells, mature erythrocytes lack nuclei and a lipid transport system. This presents a problem for the *P. falciparum* parasite because it does not have a *de novo* cholesterol synthesis pathway, but nevertheless requires cholesterol to grow and survive^19^. Overall, this suggests that *P. falciparum* acquires cholesterol from the external environment, but what type of cholesterol it is, where it comes from, and how it is transported to parasites residing in infected erythrocytes are unanswered questions at present. To help address such questions, a new imaging system capable of visualizing the internal structural alterations in living *P. falciparum*-infected erythrocytes is needed.

A variety of microscopic observations have yielded important information on the biological processes that involve structural modifications inside cells, and recently, label-free imaging, such as Raman microscopy^20–22^, focused ion beam scanning electron microscopy (called FIB-SEM)^23,24^ and optical diffraction tomography combined with holographic microscopy (holotomographic microscopy), have been developed. Notably, holotomographic microscopy, which includes phase contrast microscopy, was developed to combine holographic and tomographic techniques based on measuring the refractive index (RI) of a sample^25–28^. The best characteristic of this technique is that chemical or physical fixation is not necessary and holography can reproduce original objects with authenticity to provide three-dimensional (3D) structural information. Holographic microscopy can also identify individual cellular components because different components have different RIs. Holotomography makes it possible to observe sequential membrane migration and alterations in the structure of living cells with time-lapse information^29–31^. Here, we used time-lapse holotomography to observe how cholesterol is transported and sorted in *P. falciparum* parasitized-erythrocytes (pRBCs). Our study is the first to elucidate the sequential dynamics of membrane cholesterol transport in erythrocytes infected with live *P. falciparum* parasites.

## Holotomographic Imaging of the Non-Parasitized Erythrocyte (nRBC)

**Figure 1:**
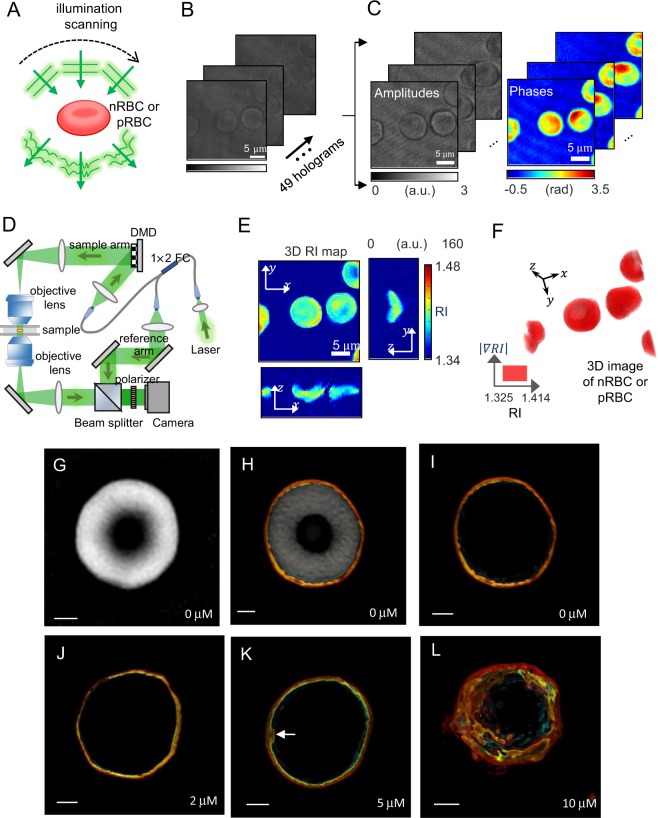
Representative 3D holotomogram based on intrinsic optical diffraction tomography (iODT) imaging of a non-parasitized erythrocyte (nRBC). (**A**) The illumination scanning process. (**B**) Acquisition of 49 holographic images of the sample with various illumination angles. (**C**) Amplitude and phase measurements of the sample. (**D**) Diagram showing the holotomographic system used in this study. DMD, digital micromirror device. (**E**) Representative 3D refractive index (RI) mapping image of the membrane and cytosol contents of nRBCs. (**F**) 3D RI mapping images of surface images of RBCs. (**G**–**I**) Representative 3D RI images of untreated nRBCs. (**J**–**L**) Representative 3D RI images of nRBCs treated with 2 μM (**J**), 5 μM (**K**), or 10 μM (**L**) concentrations of methyl-β cyclodextrin. Scale bar: 2 µm.

Holotomography, otherwise known as optical diffraction tomography, is a 3D quantitative phase imaging technique^32^. Based on the inverse scattering principle, holotomography reconstructs the 3D RI distributions of unlabeled cells and tissues from the measurements of multiple 2D holographic images^33,34^ (Fig. 1A–C). Using this technique, the 3D RI distributions of individual erythrocytes were retrieved from the tomographic reconstruction (Fig. 1D,E). For all of the images from the 3D RI tomograms of the individual erythrocytes^29,31,35,36^, we utilized a commercial optical diffraction tomographic (ODT) setup^37,38^ (Tomocube HT-2H, Tomocube, Inc., Daejeon, South Korea) (Fig. 1F). First, we examined the nRBC structure using holotomography (see Methods), the representative 3D RI tomogram of which is shown in Fig. 1G. The RI nRBC-mapped images illustrate that the fundamental structure of the erythrocyte membrane consists of three layers (i.e., an outer leaflet, inner leaflet, and cytoskeleton layer), as shown in Fig. 1(H,I) and in Supplemental Movie 1. As expected given the asymmetrical distribution of lipids between the outer and inner erythrocyte membranes^39^, the outer and inner lipid membranes have different RIs. Our tomographic images show four different RI depictions of an individual nRBC, where the areas with specific RI values were found to range from 1.331–1.353 for the outer leaflet (colored red), 1.330–1.403 for the inner leaflet (colored yellow), and 1.369–1.397 for the cytoskeleton or the protein complex (colored blue), while the remaining gray colored area is the cytosol (Fig. 1H). These results correspond with the known structural aspects of nRBC partitioning of the cytosol, membrane cytoskeleton, inner leaflet, and outer leaflet^40^.

To investigate the cholesterol localization features of the erythrocyte membrane, the RI mapping images of the nRBCs after methyl-β cyclodextrin (MβCD) (Sigma, St. Louis, IL, USA) treatment, which removes membrane cholesterol^41^, were investigated (Fig. 1I–L). We used various concentrations of MβCD and nRBCs and observed the erythrocyte membranes for any resulting alterations. The images from this experiment confirm that the inner leaflet, which is represented by a yellow circle in our mappings, contains cholesterol. Compared with the untreated nRBCs (0 µM MβCD; Fig. 1I), those treated with 2 µM MβCD lacked any notable differences (Fig. 1J). In contrast, following treatment with 5 µM MβCD, a part of the inner leaflet (yellow circle) protruded (Fig. 1K, white arrowhead) and, following treatment with 10 µM MβCD, the yellow circle representing the inner leaflet failed to maintain its inner circular form (Fig. 1L). Additionally, the outer membrane (shown in red) and membrane cytoskeleton/membrane protein complex (shown in blue) were also irregularly shaped in the nRBCs treated with 10 µM MβCD (Fig. 1L). These data confirm that holotomographic imaging can successfully reveal the multiple layers of the erythrocyte membrane. Furthermore, the changes to the inner leaflet (shown in yellow) in response to MβCD treatment are suggestive of the presence of cholesterol in the inner leaflet, and the structural aspects of the erythrocyte involving RI mapping were stable (Supplemental Movie 1) without MβCD.

## Infection-Related Objects in pRBCs Visualized by Holotomographic Imaging with RI Mapping

**Figure 2:**
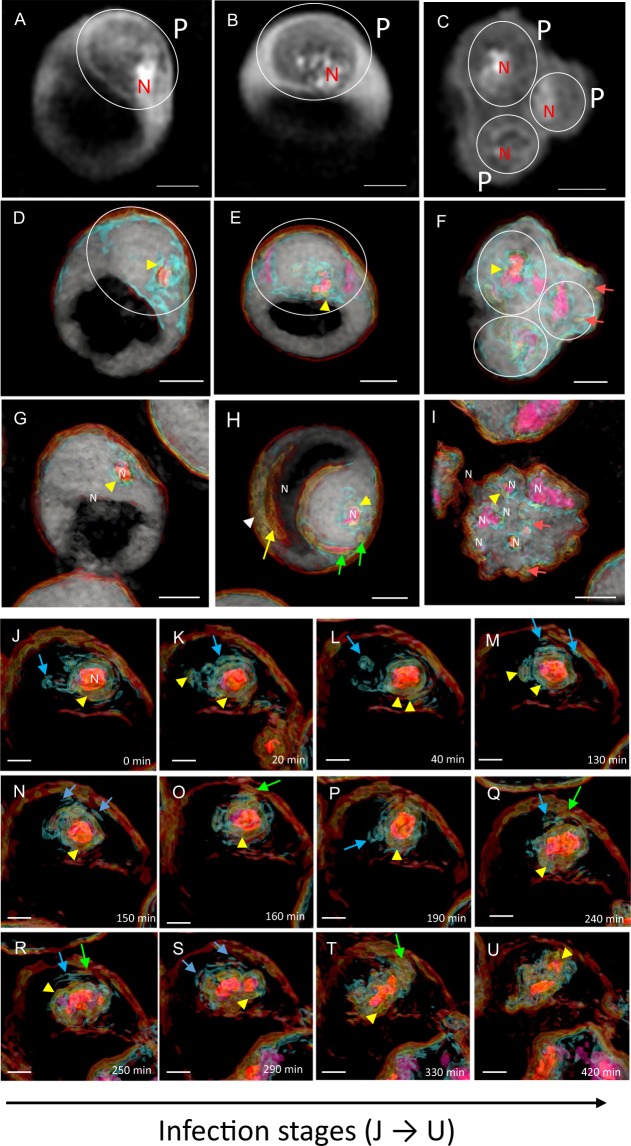
Three-dimensional holotomographic imaging of *P. falciparum*-infected erythrocytes (pRBCs). (**A**,**B**) Representative 3D holotomogram images of different trophozoite stages. (**C**) Representative 3D holotomogram image of the schizont stage. White circles (P) indicate parasites, and the high refraction area indicates parasite nuclei. (**D**–**F**) Representative 3D RI mapping of **A**–**C**. Nuclei (N) are indicated as pink–orange. (**G**–**I**) Another representative stack of pRBCs. (**J**–**U**) Representative 3D RI mapping images of pRBCs. These images were captured one by one from time-lapse observations spanning approximately 21 h. The yellow arrowheads indicate the position of the inner leaflet (yellow line) surrounding the parasitophorous vacuole membrane and/or parasite plasma membrane. The green arrows mark the place where the yellow line corresponds to the erythrocyte membrane buds. The red arrows mark small yellow vesicles/segments, and the blue arrows mark blue segments that may be fragments of the erythrocyte cytoskeleton or a protein transport carrier (presumably, for example, Maurer’s cleft or J-dots). Scale bars: 2 µm (**A**–**I**), 1 µm (**J**–**U**).

To investigate how *P. falciparum* alters the inner erythrocyte structure, we next applied 3D holotomographic imaging to pRBCs. The images captured by the 3D hologram are shown in Fig. 2(A–C), where the white circles indicate parasites (Fig. 2A–F), and the high refraction area indicates parasite nuclei (“N”). The RI ranges for the nuclei were 1.403–1.436 (pink–orange region, Fig. 2D–F).

The images shown in Fig. 2A,B,D,E were taken approximately 18–24 h after merozoite invasion, whereas those shown in Fig. 2C,F were taken approximately 36 and 38 h post-invasion, respectively. Notably, each nucleus (Fig. 2D–I, pink–orange region) is surrounded by a membranous structure that has same the RI mapping to the inner leaflet, and is thus likewise colored yellow. The outer wide membrane appears to be protruding up from the erythrocyte membrane (Fig. 2H, white arrowhead). Evidently, the red and yellow lines in these images can be seen to extend from the pRBC membrane at multiple locations and are associated with the parasite, presumably at the parasitophorous vacuole membrane (PVM) (Fig. 2H, green arrows). Cholesterol segments in the lipid membrane, which were also visualized, are represented by yellow circles (Fig. 2F,I, orange arrows). 3D RI mapping of the trophozoite stages through to the early schizont stages in live pRBCs is shown in Fig. 2J–U. In all the pRBC images, which were taken sequentially, the nuclei (orange regions) were surrounded by one RI-visualized membrane (colored yellow, marked by yellow arrowheads) or another RI-visualized membrane (colored blue, marked by blue arrows). Intriguingly, multi-layered membranes can be seen to arise from some part of the erythrocyte’s inner leaflet (represented by a yellow line) and elongate to the parasite, likely toward the PVM (Fig. 2O,Q,R,T, green arrows). Various sizes of the protein-containing region in the cytosol of the erythrocytes were apparent (Fig. 2, blue arrows). Several flat, round objects of 230–670 nm in length were also seen in the cytosol, and parts of these blue-colored objects that co-localized with the yellow membrane segments (yellow arrowheads) apparently exist in parts of the erythrocyte membrane cytoskeleton. Also, these independent flat and round objects, which were detected only in the trophozoite to schizont stages of the parasites, migrated into the cytosol (Supplemental Movie 2).

## Ezetimibe Interrupts Lipid Transport

**Figure 3:**
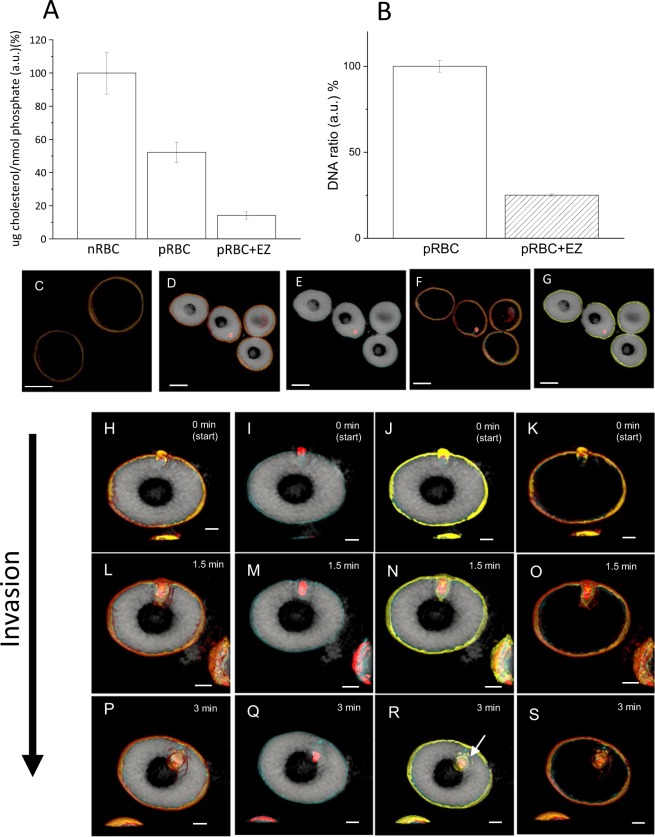
Effect of ezetimibe on *P. falciparum*-infected erythrocytes (pRBCs). (**A**) Comparison of the cholesterol to phospholipid ratio in non-parasitized erythrocytes (nRBCs), pRBCs, and ezetimibe-treated pRBCs. Y axis: a.u. denotes arbitrary units. (**B**) The effect of ezetimibe (80 μM) on *P. falciparum* growth and survival (measured as a DNA ratio; this a ratio of green fluorescence in pRBCs with vs without ezetimibe treatment). Each data point was calculated as an average of 6 identical samples and repeated 3 times. To quantitatively measure the total number of parasites in the culture we used a SYBR green assay as a proxy measure of parasite multiplication. (**C**) RI mapping of non-parasitized erythrocytes under ezetimibe treatment. There was no effect on morphology or the RI values when compared with the no treatment control. (**D**–**G**) Representative 3D RI mapping images of pRBCs that were treated with ezetimibe for 20 h. (**H**–**S**) Sequential 3D RI mapping of merozoite invasion in pRBCs after 20 h of ezetimibe treatment. Ezetimibe did not affect parasite invasion into the erythrocytes. Scale bars: (**C**–**G**) 5 µm, (**H**–**S**) 2 µm.

Cholesterol is absorbed in the human body *via* the Niemann-Pick C1-Like 1 (NPC1L1) protein, which is expressed in the small intestine^42^. Because ezetimibe has been clinically used as an inhibitor of cholesterol incorporation into cells/tissues^43–45^, this reagent is well suited for determining whether or not *P. falciparum* uses NPC1L1 for cholesterol transport. Consequently, we investigated the inner membrane structure of nRBCs and pRBCs under ezetimibe treatment. After incubation with ezetimibe, the phospholipids and cholesterol extracted from the erythrocytes were fluorometrically analyzed and the total amount of cholesterol recovered, as normalized against the total amount of phospholipid inorganic phosphate recovered, was compared between the nRBCs and pRBCs, and the results were confirmed by capillary HPLC analysis. The pRBCs contained significantly lower amounts of cholesterol than the nRBCs (Fig. 3A; *t*-test = 3.41723, Prob > l t l = 0.02685). In the presence of ezetimibe, the cholesterol amounts in the pRBCs decreased markedly compared with those of the untreated pRBCs (*t*-test = 5.84874, Prob > l t l = 0.01625). These results show that ezetimibe treatment inhibited cholesterol uptake by *P. falciparum* from outside and/or the cytosol of the parasitized erythrocytes. To assess the effect of ezetimibe treatment on parasite replication, we next monitored parasite growth in the continued presence of ezetimibe over multiple asexual cycles by SYBR green assay^46^, and ezetimibe dramatically inhibited the parasite multiplication (Fig. 3B). As successful *in vitro* culturing of *P. falciparum* requires the culture medium to contain artificial or human serum, this possibly explains why a small number of parasites were still alive after ezetimibe treatment. 3D holotomographic RI mapping of ring-stage parasites within ezetimibe-treated nRBCs and pRBCs is shown in Fig. 3C–G and Supplemental Movie 3. The morphology of the nRBCs was unaffected by ezetimibe treatment (Fig. 3C). In the presence of ezetimibe, however, we failed to detect any translocation of the lipid membrane, which included cholesterol (colored yellow) encircling the parasites, indicating that there was no additional cholesterol incorporation in these cells.

## Ezetimibe Does Not Interfere with Parasite Invasion

Assessing the effect of ezetimibe treatment on parasites further, we found cholesterol localization in the erythrocyte membranes and inner structure of pRBCs with ezetimibe as shown by RI mapping (Supplement Movie 3). However, not much is known about whether ezetimibe interferes with parasite invasion or not. Our 3D RI mapping of the active merozoite invasion process during ezetimibe treatment, as detected by time-lapse tomographic imaging, is shown in Fig. 3H–K,L–O,P–S. These imaging results clearly show that the *P. falciparum* invasion process forced the erythrocyte membrane down into the cytosol when it was inverted and, finally, the inner cholesterol-containing RBC leaflet was observed to surround the parasites as well. The three-stage invasion processes occurred over approximately 3 min. Additionally, the cytoskeleton and/or protein-containing region and the outer erythrocyte leaflet were observed to wrap around the parasite^47^ (Fig. 3L,P,N,R,O,S). We also observed small, shorter and flatter membrane segments in the cytosol of the ezetimibe-treated cells (Fig. 3R, white arrow); these did not exist under normal parasite culture conditions (i.e., without ezetimibe treatment) (Fig. 3C).

## Ezetimibe Treatment Causes Cholesterol Accumulation

**Figure 4:**
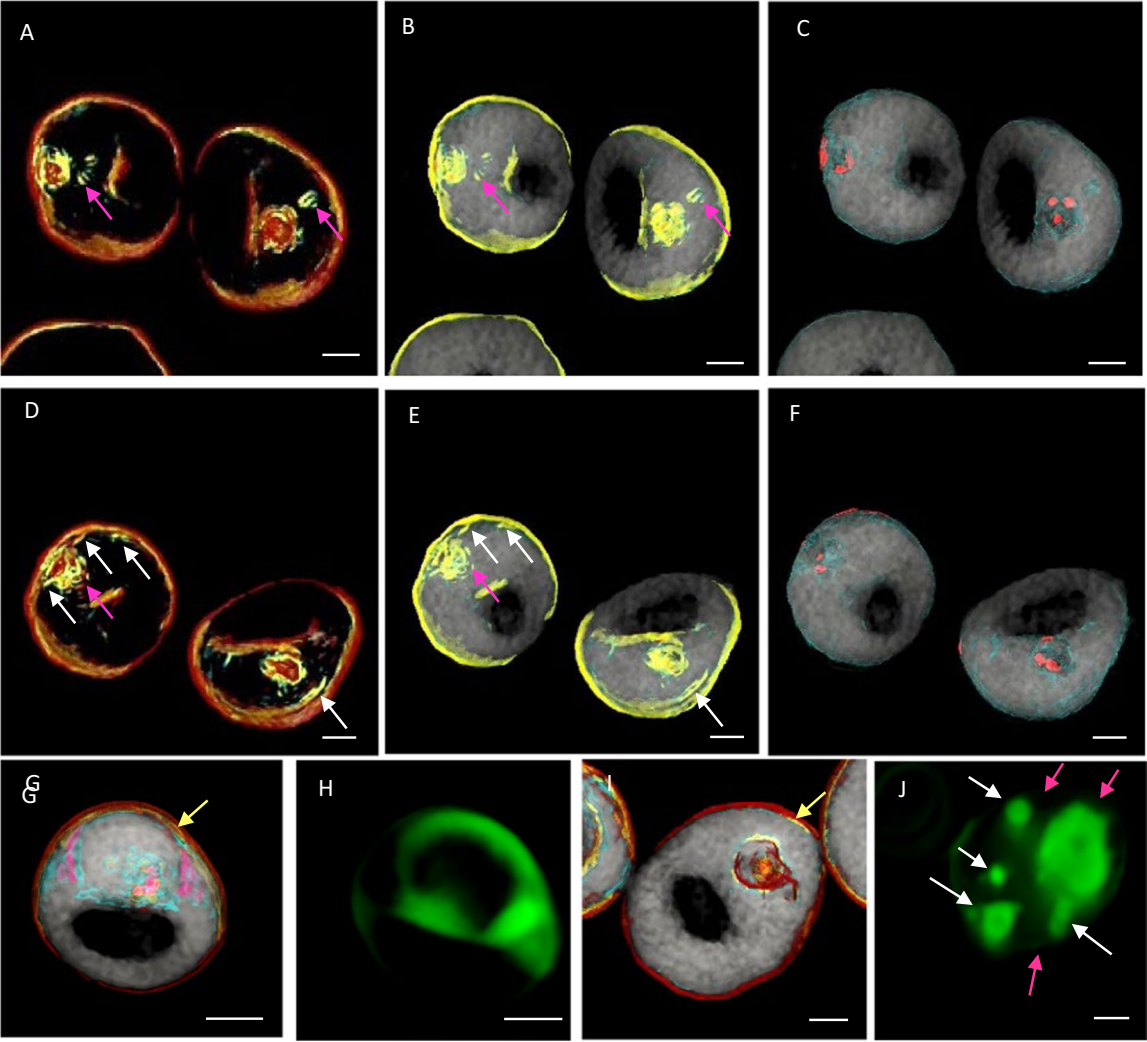
Three-dimensional RI mapping images of ezetimibe-treated *P. falciparum* parasitized-erythrocytes (pRBCs). (**A**–**F**) Images from two different cells after 40 h of 80 µM ezetimibe treatment. The pink arrows indicate small membrane segments in the cytosol and stacked outside of the parasitophorous vacuole membrane. The white arrows mark small invaginated membranes arising from the host erythrocyte membrane. (**G**,**H**) Representative images of untreated pRBCs and (**I**,**J**) pRBCs after 40 h of 80 µM ezetimibe treatment. (**H**,**J**) pRBCs were stained with bodipy-cholesterol. Cholesterol localization in the pRBCs was altered by addition of ezetimibe. Scale bar: 2 µm.

Our next experiment investigated whether ezetimibe has any influence on cholesterol transport and/or the internal membranous structure of pRBCs. During ezetimibe treatment, we observed the inner structure of the pRBCs by 3D holotomographic imaging. Small membrane segments were formed in the cytosol (Fig. 4A,B,D,E; pink arrows), and an elongated inner leaflet emerged from the erythrocyte membrane (Fig. 4D,E, white arrows), suggesting that cholesterol was not delivered to the parasites, but a small segment containing cholesterol accumulated. To verify the effect of ezetimibe on cholesterol localization in pRBCs using a different technique, we also observed pRBCs by fluorescence microscopy using bodipy-cholesterol as the fluorescence label (Fig. 4H,J) and RI mapping by way of comparison (Fig. 4G,H). However, the two different techniques did not produce co-localized results because an erythrocyte fluctuation occasionally occurred during the switch over from holographic to fluorescence image capture. Nevertheless, we still observed the same cholesterol distribution and accumulation patterns in the parasite, and presumably in the PVM also^19^, as well as in the erythrocyte membrane when ezetimibe was absent (Fig. 4G,H). In contrast, when ezetimibe is present, almost no cholesterol accumulation was seen in the pRBC membrane (Fig. 4J, pink arrows), except for a small amount (yellow arrow, Fig. 4I), and the fluorescence imaging clearly revealed that cholesterol had accumulated in the erythrocyte cytosol (i.e., it had not been sufficiently taken up by the parasite (Fig. 4J, white arrows). Collectively, these findings show that the parasite was unable to internalize cholesterol when ezetimibe was present.

## Dead Parasites have Defective Membrane Transport

**Figure 5:**
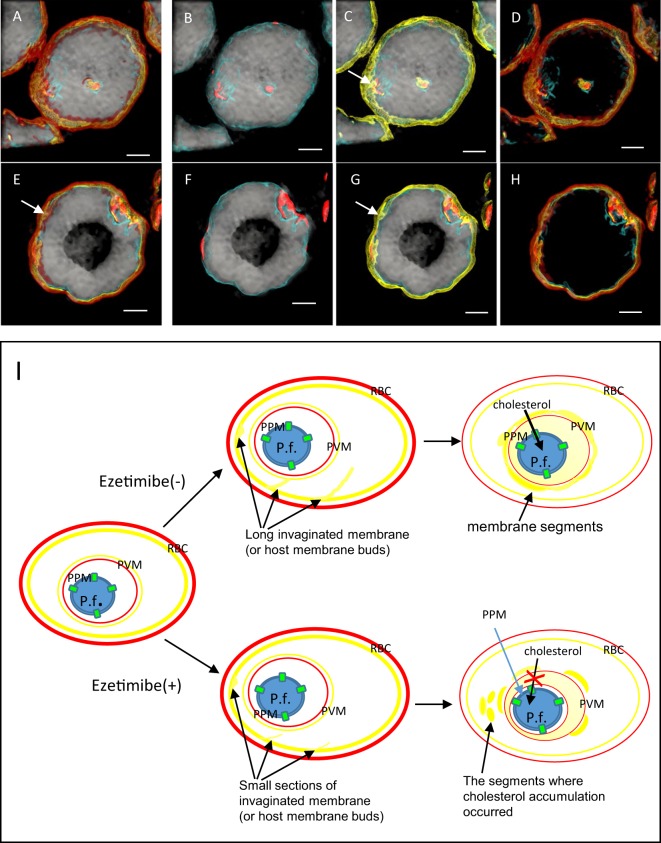
Tomographic imaging of dihydroartemisinin-treated *P. falciparum* parasitized-erythrocytes (pRBCs). (**A**–**D**) Representative images of pRBCs treated with 5 nM dihydroartemisinin (DHA) for 3 h, and (**E**–**H**) for 24 h, both at 37 °C. Membrane transport was not induced in “dead” *P. falciparum* after treatment with DHA. (**I**) Schematic summarizing the alterations observed to the intercellular structures of individual pRBCs with and without of ezetimibe treatment, focusing on the invagination, migration, and sorting of the cholesterol-containing erythrocyte membrane in the pRBCs. Scale bar: 2 µm.

Artemisinin is a widely used anti-malaria drug, and dihydroartemisinin (DHA), its degradation product, is produced in the human liver. To confirm that the observed elongated membranes and/or vesicle membranes are associated with live intraerythrocytic *P. falciparum* parasites, we examined the effects of DHA^48,49^ on a non-synchronized *P. falciparum* culture. The tomographically images of pRBCs in the presence of 5 nM DHA for 3 h (Fig. 5A–D) or for 24 h (Fig. 5E–H) at 37 °C are shown. A small inner leaflet section can be seen to elongate on the inside of each pRBC (Fig. 5, white arrows). However, there were no fragmented membranes visible in the cytosolic compartments of live intraerythrocytic *P. falciparum* parasites (Figs. 3 and 4). These results confirm that when the parasites were drug killed, membrane transport in them did not occur.

## Discussion

### Cholesterol sorting in pRBCs

Previous studies have investigated the membrane dynamics involved in lipid and protein transport in normal or pathogen-infected eukaryotic cells^50–53^. In common with its host cells, the protozoan parasite *P. falciparum* also makes various membranes. One of these membranes, a large lipid membrane known as the tubovesicular network (TVN), is formed during *P. falciparum* development, particularly during the ring through trophozoite stages^19,54^. The role of the TVN, which secretes buds from the PVM, is to transport sphingomyelin and proteins, including the raft fraction, to the erythrocyte membrane via its elongation^54,55^. This is accompanied by an increased surface area of the TVN, and also occurs at the PVM, which becomes larger as the parasite grows. For these phenomena to proceed properly, it is essential that lipids are supplied to the parasite from some source. A few studies have been conducted to determine whether *Plasmodium* parasites source cholesterol from the host erythrocyte or rely on its supply from the external environment. In one such study, Tilley *et al*. reported that lipoproteins are one source of the cholesterol that is taken up by the parasites^56^. Another study by Istvan *et al*. reported that the 3D7 strain of *P. falciparum* has *P. falciparum*-encoded NPC1L1 (PfNCR1) protein localized in the parasite plasma membrane (PPM), and that this protein is a key player in cholesterol uptake (and a potential target of antimalarial drugs)^57^. NPC1L1 is widely accepted as playing a role in the intercellular transport of cholesterol in human cells and it is possible that malaria parasites take up cholesterol into their bodies via this protein. This speculation is supported by our cholesterol assay (Fig. 3A), parasitemia assay (Fig. 3B), membrane-containing cholesterol transport video (Supplemental Movies 2 and 3) and the cholesterol accumulation images induced by ezetimibe treatment (Fig. 4). As a summary of the data from this study, Fig. 5I depicts the possible mechanism for how *P. falciparum* parasites take up cholesterol, which involves membrane transport in the cytosol of the pRBCs, with particular focus on the events likely occurring at the PPM.

Looking at membrane transport between the PVM and erythrocyte membrane from another perspective, we propose that “reverse membrane dynamics” may occur in the pRBCs in the form of a “supply and demand” process. A one-directional supply from the PVM to the erythrocyte membrane via the TVN fits with the report from Lauer *et al*.^19^, which describes lipid/protein transport by the TVN. The opposite direction, from the erythrocyte membrane to the parasite via cholesterol handling in the presumed PVM, was seen in this present study. Thus, it is possible that lipid membrane recycling is active in pRBCs.

Recent advances in microscopy have allowed researchers to gain better understanding of the physiological and biochemical phenomena occurring in real time in living cells using nano-scale observations. Electron microscopy (EM) imaging has been a particularly powerful tool in these studies, and both EM and fluorescence microscopy are convenient and well-established techniques used in biology. However, EM requires the dehydration and fixation of samples and access to an experienced technician, while fluorescence microscopy requires the use of fluorescence probes that can damage samples via phototoxicity. Thus, the target organelle or molecule in the studied cells is not in its “native” configuration during EM or fluorescence microscopy. To address these issues, we employed holotomographic imaging on live cells because it is non-invasive and does not require sample fixation; thus, use of this technique has allowed us to obtain intracellular structural information about a poorly understood aspect of *Plasmodium* biology. Despite being inferior to EM in resolution, holographic imaging is able to provide information on the structural alterations occurring over time in live cells via time-lapse imaging, something not possible with EM. Here, we visualized the membrane cholesterol/cholesterol transport system configuration in the cytosol of pRBCs by living *P. falciparum* in 3D and 4D. This convenient to use, cutting-edge technique may be helpful in revealing other biological aspects of malaria infections. One drawback of this technique was the occurrence of floating cells, something that will need to be resolved in future. It should be mentioned also that we could not observe the same individual pRBCs sequentially with time-lapse after merozoite invasion into the erythrocytes at 10–12 h post-invasion, because during that time the parasites changed into amoeba-like forms and moved around frequently in the PVM, a phenomenon known to occur in the early ring-stages^58^.

In conclusion, our unlabeled tomographic imaging results suggest that *P. falciparum* initially takes up external cholesterol and/or membrane cholesterol from the inner erythrocyte membrane, after which budding lipid membranes are elongated, migrate to the cytosol, and may fuse with and/or pass through the PVM to eventually reach the parasite body. Once this process is complete, the parasites possibly take up cholesterol via a membrane protein, such as PfNPC1, at the PPM. The mechanism involved in how membrane cholesterol passes through the PVM remains unknown; however, Tokumasu *et al*. reported that a cholesterol-rich complex moved from the pRBC membrane to the PVM^59^. In the present study, we visualized the membrane cholesterol/cholesterol transport system’s configuration in the cytosol of pRBCs. Our findings strongly support the hypothesis that there is a “membrane recycling system” between the PVM and erythrocyte membrane in pRBCs.

## Methods

### *P. falciparum* cultivation

*P. falciparum* (3D7 strain) was cultivated as previously described^5,60^. Human erythrocytes from O^+^ blood were purchased from the Japanese Red Cross Society (JRCS) (authorization number: 25J-0045). This blood was NOT from patients but from blood donation to JRCS that does not disclose personal information to us. JRCS did informed consent with donors before the blood was released to us. This study was approved by the Jichi Medical University Clinical Research Ethics Committee (authorization number 14–12 for the use of human blood samples). All methods were performed in accordance with our university’s guideline and regulations.

### MβCD, ezetimibe, and DHA treatment

MβCD^59^, ezetimibe (Cayman Chemical Company, Ann Arbor, MI, USA), or DHA (Tokyo Chemical Industry Co., Ltd., Tokyo, Japan) treatments were modified from previous reports.

### Fluorescence labelling of cholesterol

Cellular cholesterol and membrane cholesterol were both labeled with bodipy cholesterol [23-(dipyrrometheneboron difluoride)-24-norcholesterol; Avanti Polar Lipid, Inc., Alabaster, AL, USA] as previously described^59,61^.

### Lipid purification and HPLC analysis

Each sample was washed three times with TBS [20 mM Tris-HCl, 150 mM NaCl, pH 7.4]. The trophozoite and schizont stages in pRBCs (packed cells) were separated by Percoll PLUS (GE Healthcare, Uppsala, Sweden), followed by MACS Separators LS column separation (Miltenyi Biotec K.K., Bergisch Gladbach, Germany). Lipids were extracted^62^ and then dried using N_2_-gas to produce a dry lipid film in glass tubes. Cholesterol was quantitated using cholesterol oxidase and a coupled fluorescent substrate (Sigma-Aldrich) in a Tecan M200 plate reader. Inorganic phosphates were measured from the total phospholipids recovered using the Malachite Green assay with aliquots of the lipid preparations in chloroform/methanol (1/1, vol/vol). Samples were dried under N_2_-gas in glass vials, and 0.5 mL of 6 M HCl was added to the vials, which were sealed and incubated for 8 h at 150 °C. The solvent was vacuum-dried, the residue dissolved in water, and then assayed for phosphate content. The samples were also analyzed on an Agilent 0.5 × 150 mm SB C_18_ capillary column at a flow rate of 12 µL/min, acetonitrile/tetrahydrofuran/water, 65/35/7, gradient to no water, with detection and integration at 215 nm.

### SYBR green assays

SYBR green assays were performed by modifying the methods from previous studies^46,63,64^ for evaluating *P. falciparum* growth. Briefly, *P. falciparum* was cultured for ≈96 h (two cycles), the *P. falciparum*-RBC burst was collected in sterilized water, and the erythrocyte membranes were removed by centrifugation (18,000 × g, 15 min). Aliquots (100 μL) of the supernatant from each sample were mixed with 100 μL of the assay reagents; SYBR green (Molecular Probes Inc., Eugene, OR, USA), Triton X-100 (Roche Diagnostics GmbH, Mannheim, Germany), saponin (Sigma), 20 mM-Tris (Invitrogen) and 500 mM EDTA (Dojindo, Kamimashiki-gun, Japan) for 1 h at room temperature. The fluorescence intensity of the SYBR G was measured by Molecular Devices Spectra Max M5 (Molecular Devices, LLC., San Jose, CA, USA) (EX = 521 nm, EM = 591 nm).

### Holotomography

Detailed information about the system and reconstruction algorithms we used can be found elsewhere^37,65,66^. Briefly, the ODT setup was based on an off-axis Mach-Zehnder interferometer equipped with a digital micromirror device (DMD). This is where a beam from a coherent, monochromatic laser (wavelength, λ = 532 nm) is split into a sample and a reference arm, using a 2 × 2 single-mode fiber coupler. The DMD is exploited to control the angle of the beam impinging onto the sample^67,68^. The light diffracted from the sample is collected using an objective lens (60×, numerical aperture = 1.3) and a tube lens (f = 175 mm). The sample beam is combined with the reference beam by a beam splitter, after which the spatially modulated hologram is recorded by a CMOS image sensor. For the tomographic reconstruction, 49 holograms of the sample were acquired with various illumination angles. Using a phase retrieval algorithm^69,70^, the amplitude and phase images were retrieved from the measured holograms. Based on the Fourier diffraction theorem with Rytov approximation^71^, the 3D RI tomogram of the sample was reconstructed from the retrieved amplitude and phase images by TomoStudio (Tomocube, Inc.). The theoretical lateral and axial optical resolutions of the ODT system we used were 110 nm and 360 μm, respectively, according to the Lauer criterion^72^.

## Supplementary information

Supplementary Movie 1.

Supplementary Movie 2.

Supplementary Movie 3.
